# Supplementary material for: Coupling a single solid-state quantum emitter to an array of resonant plasmonic antennas
Source: Sci Rep. 2018 Feb 21;8:3415. doi: 10.1038/s41598-018-21664-8 (PMC5821882; doi:10.1038/s41598-018-21664-8)
Supplement: Supplementary file 1 — Supplementary information [file 41598_2018_21664_MOESM1_ESM.pdf]

Supplementary information: Coupling a single solid state quantum emitter to an array of resonant plasmonic antennas

Markus Pfeiffer,[†,‡,¶] Paola Atkinson,[§,‖] Armando Rastelli,[§] Oliver G. Schmidt,[§] Harald Giessen,[¶] Markus Lippitz,[*,⊥,‡,¶] and Klas Lindfors[*,†,‡,¶]

†*Department of Chemistry, University of Cologne, Luxemburger Str. 116, D-50933 Köln*

‡*Max Planck Institute for Solid State Research, Heisenbergstrasse 1, D-70569 Stuttgart*

¶*Fourth Physics Institute and Research Center SCOPE, University of Stuttgart, Pfaffenwaldring 57, D-70550 Stuttgart, Germany*

§ *Institute for Integrative Nanosciences, IFW Dresden, Helmholtzstrasse 20, D-01069 Dresden, Germany*

‖*Sorbonne Universites, UPMC Univ Paris 06, CNRS, UMR 7588, Institut des Nanosciences de Paris, 4 place Jussieu, F-75252 Paris, France*

⊥ *Experimental Physics III, University of Bayreuth, Universitätsstrasse 30, D-95447 Bayreuth, Germany*

E-mail: markus.lippitz@uni-bayreuth.de; klas.lindfors@uni-koeln.de

Experimental setup

All optical measurements are performed in a home-built laser-scanning confocal microscope. This setup can also be used to perform detection-spot scanning dark-field micro-spectroscopy, as illustrated in Fig. S1 a). For plasmonic antennas on semiconductor substrates it is nec-

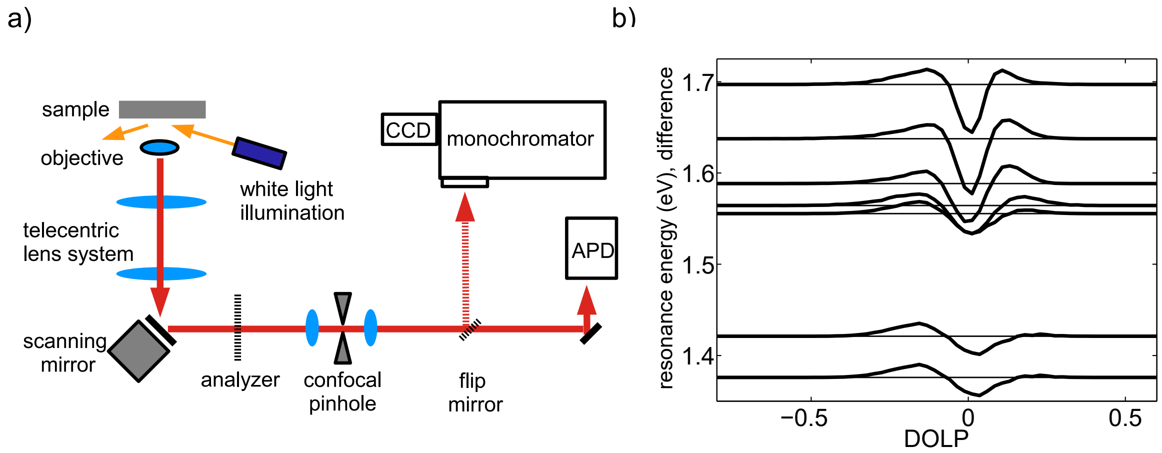

Figure S1: a) Experimental setup. b) Difference of histograms for reference emitters and emitters beneath arrays with the indicated resonance energy.

essary to have control over the polarization state of the illumination to be able to distinguish between the different modes. To achieve this, a fiber-coupled illumination module was designed and implemented in a home-built laser-scanning confocal microscope. The illumination source is a 100 W halogen lamp that is coupled into a large core multi-mode fiber using a hemispherical lens with a focal distance of 20 mm. The light exiting the fiber is collimated, polarized with a broadband polarizer, and then slightly focused on the sample under an adjustable angle. Light scattered by an optical antenna is collected by the objective in the laser-scanning confocal microscope, passed through the confocal pinhole, and then directed either onto a single photon counting avalanche photodiode (APD) module or to a spectrometer equipped with a charge coupled device camera (CCD). The sample area from which light is detected can be controlled with the scanning mirror in the microscope.

Details on quantifying the excess DOLP

To quantify the change in the DOLP distributions we calculate the difference of the normalized histogram for the antenna array N_{res} and the unpatterned quantum dot region N_{ref}. The photoluminescence signals of the quantum dots for both polarizations are spectrally integrated intensities from 1.570 eV to 1.656 eV. The differences of the histograms for all

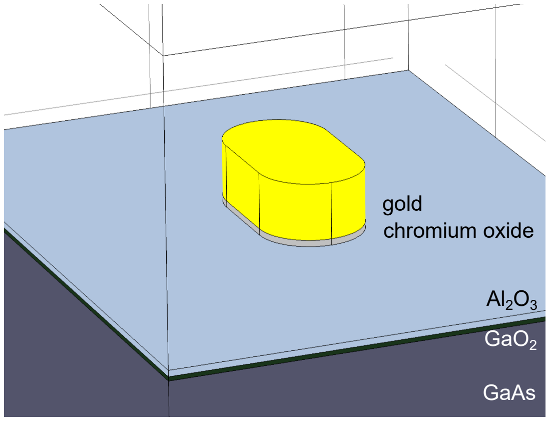

Figure S2: Structure used for simulations

antenna arrays are displayed in Fig. S1b). To quantify the changes due to the arrays we calculate the centers of mass for the differences of the histograms. We perform this analysis separately for positive and negative values of DOLP. This allows us to observe changes in the shapes of the histograms in order to gain insight into how the enhancement or suppression of emission depends on the properties of the plasmonic array. For positive values of DOLP we define the DOLP excess as

$$m^{+} = \int_{0}^{1} (N_{\text{res}} - N_{\text{ref}})\,\text{DOLP}\;\text{d(DOLP)}. \tag{1}$$

For negative values of DOLP we define

$$m^{-} = \;-\int_{-1}^{0} (N_{\text{res}} - N_{\text{ref}})\,\text{DOLP}\;\text{d(DOLP)}. \tag{2}$$

Finite element simulations

Simulations are performed with a finite element solver (Comsol Multiphysics with RF module, Comsol Ab, Stockholm, Sweden) for frequency domain electromagnetic full field calculations. As described in previous publications,[1] we apply the Lorentz reciprocity theorem to

deduce the emitted power from oriented electric point dipoles[2] at the position of the quantum dots. The periodicity is included by applying Floquet periodic boundary conditions on the side boundaries of the arrays' unit cells. For the calculations of the intensity distribution around an isolated single antenna, we apply a two step simulation. First, the incident fields are calculated, while the material of the antenna is set to vacuum. In a second step, the field of the first simulation step is taken as the incident field, the antenna is modeled as a nanorod of gold and chromium oxide, and periodic boundary conditions are replaced by perfectly matched layer (PML) domains with outer scattering boundary conditions.

The structure is modeled according to dimensions determined from scanning electron micrographs and atomic force microscopy measurements. The antennas are rectangular gold nanorods of 90 nm length, 60 nm width, and 27 nm height. The dielectric constants of gold are interpolated from Ref.[3] Beneath the antennas there is a 3 nm thick layer of chromium oxide (refractive index = 2.5). The substrate consists of an infinite substrate halfspace with a refractive index of 3.5 on top of which there is a 2 nm thick native gallium oxide layer (refractive index = 1.9).[4] At the sample surface the 3 nm thick Al_2O_3 passivation layer is modeled as a layer with a refractive index of 1.76. To adapt the element shapes to those determined for similarly fabricated gold structures from transmission electron micrographs,[1] the side corners of the antenna elements are rounded with a radius of 25 nm (see Fig. S2). From transmission electron micrographs we determine a distance of 21 nm between the center of the quantum dots and the top of the substrate. Thus, in the simulations we extract the intensity distributions for two orthogonal polarization states at this distance from the substrate air interface.

Influence of the array period

Here we analyze, how the observed mode is influenced by the period of the plasmonic array. For this, we keep the emission wavelength constant at the QD emission ($\lambda_{0,QD} = 760$ nm)

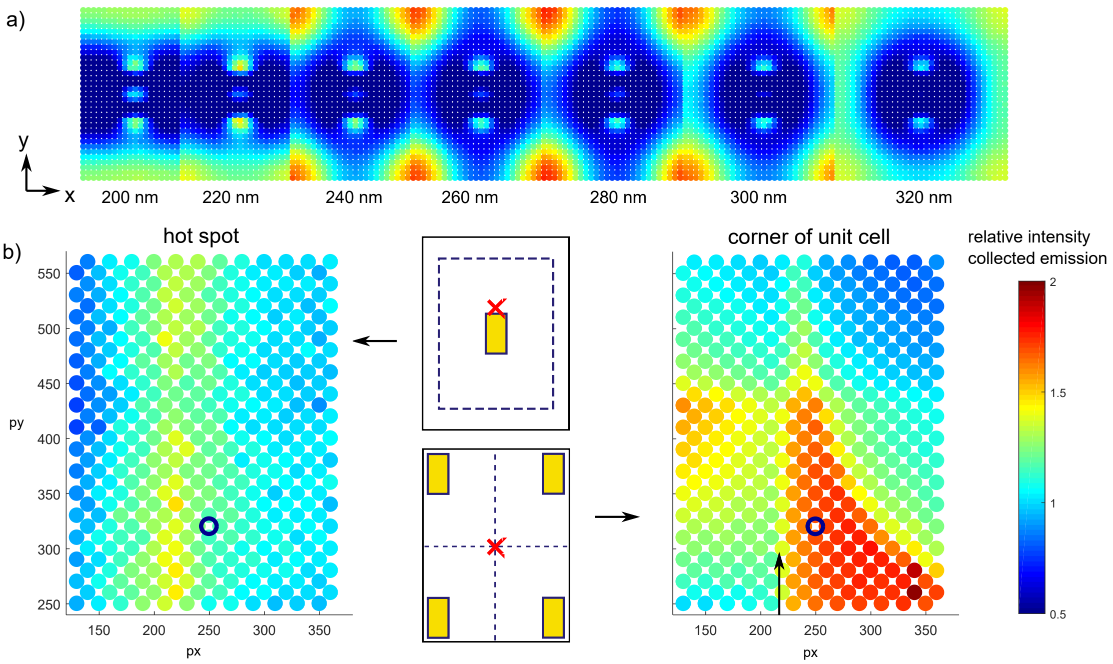

Figure S3: a) Intensity distribution for a y-oriented dipole in the plane of the quantum emitters. The period in y-direction is kept constant (320 nm) while the period in x-direction is varied from 200 nm to 320 nm. b) Enhancement of detected emission for different array periods for a dipole located at a distance of 10 nm from the tip of the antenna elements (left) and in the corner of the unit cell (right). The vertical arrow marks the array period p_x where the first diffraction order becomes radiative into the substrate. The arrays studied experimentally in this work are marked with a blue circle.

and investigate the collected intensity of a y-oriented dipole, which is located in the plane of the quantum dots. For a constant period in y-direction (chosen identical to the experimental conditions, $p_y = 320$ nm) the intensity of the y-oriented dipole is shown as a function of its lateral position in the unit cell [see Fig. S3a)]. One observes, that there are regions of enhanced emission close to the ends of the antenna elements and in the corners of the unit cell. For these two locations we extract the relative enhancement compared to an emitter in the same environment but without the plasmonic array. This is shown in Fig. S3b). The enhancement for the quantum placed in the corner of the unit cell [see right side of Fig. S3b)] decreases for increasing period in x-direction p_x. For a period p_x of approximately 220 nm one can observe a discontinuity in the enhancement. This is due to the first diffraction order into the substrate becoming radiative as the condition $p_x > \lambda_{0,QD}/n_{substrate}$ is fulfilled.

The deviations between the spatially dependent enhancement of experiment and simulations are mainly the lack of enhancement for the y-oriented dipole at the hot-spots of the antenna elements. We attribute this to quenching of the emission due to an additional absorption rate due to the proximity of the metal structure. This additional rate is not considered in our simulations. Another reason are geometrical mismatch between the size of the hot-spots and the lateral dimension of our quantum dots. The spatial averaging of the enhancement pattern results in a decrease of the calculated maximum values, where we considered point dipoles. Particularly, from Fig. S3 it becomes obvious, that the enhancement shows a different dependence on the lateral array periods than the hot-spots close to the long ends of the array elements.

References

(1) Pfeiffer, M.; Lindfors, K.; Zhang, H.; Fenk, B.; Phillipp, F.; Atkinson, P.; Rastelli, A.; Schmidt, O. G.; Giessen, H.; Lippitz, M. *Nano letters* **2014**, *14*, 197–201.

(2) Carminati, R.; Nieto-Vesperinas, M.; Greffet, J.-J. *J. Opt. Soc. Am. A* **1998**, *15*, 706–712.

(3) Johnson, P. B.; Christy, R. W. *Phys. Rev. B* **1972**, *6*, 4370.

(4) Rebien, M.; Henrion, W.; Hong, M.; Mannaerts, J. P.; Fleischer, M. *Appl. Phys. Lett.* **2002**, *81*, 250.
